# Prophylactic Pegfilgrastim Versus On-Demand Filgrastim During Docetaxel and Cyclophosphamide Chemotherapy in Early-Stage Breast Cancer: A Retrospective Analysis

**DOI:** 10.7759/cureus.92409

**Published:** 2025-09-15

**Authors:** Hirona Banno, Kimihito Fujii, Wataru Ohashi, Kanna Ozaki, Masayuki Saitou, Mirai Ido, Manami Gotou, Andou Takahito, Junko Kousaka, Shogo Nakano

**Affiliations:** 1 Department of Surgery, Aichi Medical University, Nagakute, JPN; 2 Division of Biostatistics, Clinical Research Center, Aichi Medical University, Nagakute, JPN

**Keywords:** breast cancer, docetaxel-cyclophosphamide chemotherapy, febrile neutropenia, pegfilgrastim, relative dose intensity

## Abstract

Background and aim: In patients with early-stage breast cancer, myelosuppressive chemotherapy, such as docetaxel and cyclophosphamide (TC) chemotherapy, is frequently introduced as an adjuvant treatment postoperatively. This regimen commonly causes febrile neutropenia (FN); therefore, the use of pegfilgrastim, a newly developed granulocyte colony-stimulating factor (G-CSF), plays an important role in preventing the occurrence of FN. We evaluated the clinical advantage of pegfilgrastim during TC chemotherapy by comparing it with conventional filgrastim.

Patients and methods: Overall, 85 patients with stage I or II breast cancer who received TC chemotherapy were retrospectively analyzed by collecting data from the patient charts. The enrolled patients were divided into two groups: patients who received prophylactic pegfilgrastim administration, 36 (42.4%) (PEG(+)), and those who received chemotherapy only with the contemporary G-CSF agent, filgrastim, which was administered when they suffered from FN or severe neutropenia, 49 (57.6%) (PEG(-)). We evaluated the effectiveness of the use of pegfilgrastim for the prevention of FN, maintaining a high relative dose intensity (RDI) (first endpoint), and explored the effect of the use of pegfilgrastim on disease-free survival (second endpoint). For statistical analyses, Fisher’s exact test was applied mainly to categorical variables. The ratios of adverse event frequency in the PEG(-) and PEG(+) groups were calculated and compared. The Mann-Whitney U test was employed for the RDI of docetaxel and cyclophosphamide. The DFS rate of the PEG(-) and PEG(+) groups was estimated using the Kaplan-Meier method and compared using the log-rank test.

Results: The number of patients diagnosed as Grade 3 to 4 “neutrophil count decrease” according to the Common Terminology Criteria for Adverse Events version 5.0 was 21 (42.8%) and six (16.7%) in the PEG(-) and PEG(+) groups, respectively (P = 0.0238). The odds ratio for the onset of Grade 3 to 4 “neutrophil count decrease” in the PEG(+) group compared to that in the PEG(-) group was 0.1143 (95% confidence interval, 0.0175-0.7446). The difference in disease-free survival rates could not reach a significant level because the number of events was small.

Conclusion: The administration of pegfilgrastim significantly reduced the risk of FN development, with acceptable adverse events. The chemotherapy RDI of patients who received prophylactic pegfilgrastim was significantly higher than that of those who were administered conventional filgrastim at the physician’s discretion. In the present study, we verified the clinical efficacy of pegfilgrastim in patients receiving TC chemotherapy. The prophylactic use of pegfilgrastim is considered to be essential for carrying TC chemotherapy safely.

## Introduction

In the 1990s, doxorubicin and cyclophosphamide (AC) chemotherapy was the gold standard adjuvant chemotherapy regimen for early-stage breast cancer, although cardiotoxicity induced by doxorubicin was a concern for physicians [1,2]. Subsequently, four cycles of docetaxel and cyclophosphamide (TC) chemotherapy became the next strategy as postoperative adjuvant chemotherapy for early-stage breast cancer because TC chemotherapy produced superior disease-free and overall survival compared to four cycles of AC chemotherapy [3,4]. In a randomized trial comparing TC chemotherapy with AC chemotherapy in 1,016 patients with stage I-III breast cancer who underwent complete resection, the toxicities associated with chemotherapy were similar between the two regimens.

Doxorubicin in the AC chemotherapy regimen is cardiotoxic; however, only a single patient with congestive heart failure has been treated with AC chemotherapy (none with TC chemotherapy) [3]. The most frequent adverse event is febrile neutropenia (FN). Grades 3 to 4 neutropenia in patients aged over 65 years has been confirmed in 60% and 54% of patients on TC and AC chemotherapy, respectively. The incidence of FN is slightly higher in TC than in AC chemotherapy, without significance, whereas bone marrow toxicity and cardiotoxicity over seven years were low, demonstrating that TC chemotherapy is a feasible treatment [4].

However, racial and physical differences in abutment-tumor effects and adverse events of chemotherapy should be considered. In a feasibility study of TC chemotherapy in Japanese patients with breast cancer, Grades 3 to 4 neutropenia and FN were observed in 98.1% and 28.3% of patients, respectively. Therefore, TC chemotherapy is tolerable in Japanese patients, although attending physicians should pay attention to the development of FN and neutropenia [5].

In clinical settings, physicians are encouraged to plan and administer chemotherapy at the recommended dosages and durations. Therefore, the onset of FN should be avoided during chemotherapy. The newly developed granulocyte colony-stimulating factor (G-CSF), pegfilgrastim, which is a pegylated form of filgrastim with a long half-life in circulation, has been administered prophylactically to reduce the risk of FN. This agent is safe and significantly reduces the incidence of FN in TC chemotherapy regimens [6].

The evaluation of dose intensity and adverse events in consecutive patients at a single institution with or without pegfilgrastim administration has not been reported. Additionally, a comparative investigation of laboratory trends during TC chemotherapy in this setting has not been previously performed. We, therefore, aimed to retrospectively assess the clinical advantages of pegfilgrastim in patients receiving TC chemotherapy. Although this study consists of retrospective analyses, the patients' backgrounds for comparison are almost uniform. Moreover, the clinical cases who was affected with FN were presented in detail. These real-world data may improve supportive therapy for patients with breast cancer receiving TC chemotherapy

This article was previously posted to the Research Square preprint server on February 14, 2024 (DOI: 10.21203/rs.3.rs-3933316/v1).

## Materials and methods

Patients

A total of 1,817 patients underwent breast cancer surgery between November 2010 and November 2020 at Aichi Medical University Hospital (Nagakute, Aichi, Japan). Among them, postoperative adjuvant chemotherapy was administered in 410 (22.5%) patients, with regimens including anthracycline followed by taxane, anthracycline followed by taxane with anti-human epidermal growth factor receptor type 2 (HER2), TC, monotherapy of paclitaxel, and others, in 179 (43.6%), 104 (25.4%), 86 (21.0%), 34 (8.3%) and seven (1.7%) patients, respectively. The 86 patients undergoing the TC regimen included those who received four-cycle TC chemotherapy. One patient was excluded as chemotherapy was suspended after the second administration due to severe fatigue and nausea. A total of 85 patients undergoing TC were enrolled in this study.

Chemotherapy

TC chemotherapy consisted of the administration of docetaxel (75 mg/m^2^) and cyclophosphamide (600 mg/m^2^) intravenously for about 90 minutes every three weeks. All patients received four cycles of chemotherapy with a two-week delay. Dexamethasone (8 mg/day) was administered orally to counteract the side effects from the day before chemotherapy (day 0) until day 3, excluding day 1. On day 1, 1 mg granisetrone and 6.6 mg dexamethasone were administered intravenously as premedication for chemotherapy. None of the patients received prophylactic antibiotics during the four cycles of chemotherapy.

Complications and toxicities

If critical adverse events, which were classified as Grade 3 or higher according to Common Terminology Criteria for Adverse Events(CTCAE) version 5.0, occurred during TC chemotherapy, the dosage of each drug was reduced by 20% in the next cycle. Dose reduction was allowed twice for a 40% reduction compared to the standard dosage. Especially for Grade 2 FN or neutropenia, subcutaneous injection of filgrastim (75 mg) was commonly administered until October 2014, or the prolongation of the washout period was introduced at the physician’s decision. After the approval of subcutaneous injection of pegfilgrastim (3.6 mg) for prophylactic use to avoid FN during chemotherapy in November 2014 by the Ministry of Health, Labour and Welfare, Japan, pegfilgrastim was administered prophylactically on day 3 during TC chemotherapeutic treatment. The collection of adverse symptoms, such as nausea, anorexia, and fatigue, was carried out by evaluating the medical record documentation.

Relative dose intensity (RDI)

The RDI was defined as the ratio of the actual dose intensity (ADI) to the standard dose intensity (SDI). The formula for calculating RDI is as follows: ADI = actual dose/actual time; SDI = standard dose/standard time; RDI = ADI/SDI.

The “actual dose” is the total amount of administered chemotherapeutic agents, the “actual time” is the total time length of chemotherapy, and the “standard dose” and “standard time” correspond with the standardized programmed dose and time of chemotherapy, respectively. The RDI was calculated for both docetaxel and cyclophosphamide separately and then averaged across the regimen. In this study, calculations of the RDI for the two agents were performed on all enrolled patients because all patients received four cycles of TC chemotherapy.

Study design

Following approval from the Institutional Review Board of Aichi Medical University (No. 2022-231), de-identified patient data were retrospectively collected from the medical records. We first assembled the following factors: operative procedure, TNM stage [7], pathological diagnoses including hormonal (estrogen and progesterone) and HER2 status, administered dose and duration required of four-cycle chemotherapy, results of laboratory tests, adverse events according to the CTCAE, and post-chemotherapeutic patient status, including survival rate without recurrence. We divided the patients into two groups: patients who received prophylactic pegfilgrastim administration to prevent FN during four cycles of chemotherapy (PEG(+)) and patients who received chemotherapy only with the contemporary granulocyte colony-stimulating factor (G-CSF) agent, filgrastim, which was administered when they had suffered from FN and/or severe neutropenia (named PEG(-)).

All PEG(+) patients, excluding one who had received pegfilgrastim administration from the second cycle of chemotherapy, received prophylactic pegfilgrastim injection from the initial to the fourth cycles continuously without considering whether the patient experienced FN and/or severe neutropenia. Between the two groups, we compared the clinical data, RDI of each chemotherapeutic agent, and disease-free survival (DFS) rate after surgery. A retrospective study method was used to evaluate the effectiveness of pegfilgrastim in preventing FN and maintaining a high RDI during TC chemotherapy, and to explore the effect of pegfilgrastim on the DFS of patients.

The last day of confirmation of the patient’s status was fixed as May 31, 2022. Generally, FN is defined as a condition marked by fever over 38.3°C and a decreased neutrophil count under 500 /μL or under 1,000 /μL with a suspicion of rapid decrease within 48 h under 500/μL [8]. Severe neutropenia conformed to “neutrophil count decrease” according to CTCAE version 5.0, which is graded as follows: Grade 1, <1,500 /μL; Grade 2, 1,000-1,500/μL; Grade 3, 500-1,000 /μL; and Grade 4, < 500/μL. These findings are based on laboratory test results indicating a decrease in the neutrophil count in blood specimens.

This study was approved by the Institutional Review Board of Aichi University Hospital, named "Aichi University Hospital Institutional Review Board." The committee reference number is 2022-231.

Statistical analyses

Bell Curve for Excel (Social Survey Research Information, Tokyo, Japan) was used for the statistical analyses. The significance level was set at P < 0.05. Categorical variables were analyzed using Fisher’s exact test. Student’s t-test was applied for the comparison of the age. The ratios of adverse event frequency in the PEG(-) and PEG(+) groups were calculated and compared. A two-way analysis of variance was used to assess the laboratory test results for each cycle of chemotherapy. The Mann-Whitney U test was employed for the RDI of docetaxel and cyclophosphamide, because the sample distribution of RDI was not normal. The DFS rate of the PEG(-) and PEG(+) groups was estimated using the Kaplan-Meier method and compared using the log-rank test. If there are missing records, the data were excluded from the statistical processing.

## Results

Patient characteristics

Between the two groups, no statistical differences were confirmed in age at the time of surgery, pathological diagnoses including hormonal receptors, HER2 status, Ki67 index, or operative procedure. There were statistically significant differences in TNM classification between the two groups; however, these differences were not considered to affect the clinical course during TC chemotherapy (Table 1).

**Table 1:** Patient characteristics * Values are presented as the mean with standard deviation. Student’s t-test was used for statistical evaluation. Fisher's exact test was applied for the other categorical variables. †: Those p-values were considered not to influence the endpoints of this study. A single hyphen “-“ was inserted in the empty cells. The figures in parentheses refer to % in each group. ‡: Values represent the 95% confidence interval.

	PEG(-)	PEG(+)	P- value
Number of patients	49	36	-
Age at the operation (y/o)*	51.6 ± 11.8 (48.2-55.0)^‡^	51.7 ± 9.7 (48.5-54.9)^‡^	0.948
Pathological diagnosis	-	-	-
Invasive ductal carcinoma	43 (87.8)	34 (94.4)	0.693
Invasive lobular carcinoma	4 (8.2)	1 (2.8)	
Mucinous carcinoma	1 (2.0)	1 (2.8)	
Invasive micropapillary carcinoma	1 (2.0)	0	
Hormone and HER2 status	-	-	-
ER(+), PgR(+), HER2 (-)	43 (87.8)	33 (91.7)	0.847
ER(+), PgR(-), HER2 (-)	5 (10.2)	3 (8.3)	
ER(-), PgR(-), HER2 (-)	1 (2.0)	0	
Ki67 index (%)*	34.0 ± 15.2 (27.2-37.9)^‡^	29.5 ± 13.1 (20.7-33.4)^‡^	0.139
TNM classification	-	-	-
T category	-	-	-
T1	18 (36.7)	21 (58.3)	0.029^†^
T2	31 (63.3)	14 (38.9)	
T3	0	1 (2.8)	
N category	-	-	-
N0	37 (75.5)	17 (47.2)	0.010^†^
N1	12 (24.5)	18 (50.0)	
N2	0	1 (2.8)	
Stage	-	-	-
I	24 (49.0)	9 (25.0)	0.032^†^
II	25 (51.0)	25 (69.4)	
III	0	2 (5.6)	
Operative procedure	-	-	-
Total mastectomy	29 (59.2)	27 (75.0)	0.140
Partial mastectomy	20 (40.8)	9 (25.0)	

Table 2 shows the age distributions of the two groups. Neither the mean age nor the age distribution showed a statistically significant difference between the two groups. This indicates that these factors did not influence the chemotherapeutic course or onset of adverse events. The patients' general performance statuses in the PEG(-) and PEG(+) groups were almost equal. Although TC chemotherapy is commonly applied to hormone-positive and HER2-negative patients [9], the PEG(-) group included one hormone-negative and HER2-negative patient (Table 1).

**Table 2:** Age distribution of the patients enrolled The age ranges of patients in the PEG(-) and PEG(+) groups were 31.2–72.4 and 36.8–69.8 (y/o), respectively. No statistically significant differences were observed (P = 0.107). Fisher's exact test was used.

	Age distribution				
	30s	40s	50s	60s	70s
PEG(-)	9 (18.4)	15 (30.6)	9 (18.4)	12 (24.5)	4 (8.1)
PEG(+)	2 (5.6)	18 (50.0)	7 (19.4)	9 (25.0)	0

Adverse events

The major adverse events are summarized in Table 3. The number of patients who were diagnosed as having a Grade 3-4 “neutrophil count decrease” was 21 (42.8%) and six (16.7%) in the PEG(-) and PEG(+) groups, respectively (P = 0.0238). The odds ratio for the onset of Grade 3-4 “neutrophil count decrease” in the PEG(+) group compared to that in the PEG(-) group was 0.1143 (95% confidence interval, 0.0175-0.7446).

**Table 3:** Summary of adverse events *; The number of the patients who were diagnosed as having a Grade 3–4 “neutrophil count decrease” of the PEG(-) group was larger than that of the PEG(+) group. Statistical analysis could not be performed for the symptoms of neuralgia, dysgeusia, and edema because these categories included zero factors in both the PEG(-) and PEG (+) groups. For the other categorical variables, Fisher's exact test was applied. N.A.: not available

	PEG(-)		PEG(+)		-
Symptoms	Grades 1 and 2	Grades 3 and 4	Grades 1 and 2	Grades 3 and 4	P-value
Neutrophil count decreased	2 (4.1)	21 (42.9)	5 (13.9)	6 (16.7)	0.0238*
Fever	11 (22.4)	0	5 (13.9)	0	N.A.
Neuralgia	19 (38.8)	0	25 (69.4)	0	N.A.
Rash acneiform	35 (71.4)	2(4.1)	29 (80.6)	0	0.4998
Nausea	15 (30.6)	0	17 (47.2)	1 (2.8)	1.0000
Diarrhea	16 (32.3)	0	20 (55.6)	1 (2.8)	1.0000
Anorexia	7 (14.3)	1 (2.0)	10 (27.8)	1 (2.8)	1.0000
Arthralgia and myalgia	14 (28.6)	0	3 (8.3)	1 (2.8)	0.2222
Dysgeusia	13 (26.5)	0	16 (44.4)	0	N.A.
Fatigue	32 (65.3)	0	18 (50.0)	1 (2.8)	0.3725
Edema	20 (40.8)	0	20 (55.6)	0	N.A.

The number of patients who were hospitalized for treatment of adverse symptoms was 7(14.3%) and 1(2.7%) in the PEG(-) and PEG(+) groups, respectively (P = 0.0726) (Table 4). Admission was decided upon by the attending physician. The number of patients experiencing a high fever over 38.0°C was five (10.2%) and one (2.7%) in the PEG(-) and PEG(+) groups, respectively.

**Table 4:** Summary of the patients who were hospitalized *Days from the start (day 1) of chemotherapy. †Data on admission. Case 7 in the PEG(-) group was hospitalized every time chemotherapy was administered. The median RDIs of docetaxel and cyclophosphamide in the PEG(-) group were 74.73 and 75.21, respectively.

Group	Case number	Symptoms	Days since chemotherapy*	WBC count^†^	Hospital stays	RDI		Prognosis
						Docetaxel	Cyclophosphamide	
PEG(-)	1	Fever > 38℃	9	1100	4	82.94	82.94	Alive
	2	Nausea, arthralgia, anorexia	4	8800	2	79.62	79.81	Alive
	3	Fever > 38℃	8	900	5	65.83	66.52	Alive
	4	Fever > 38℃	9	600	6	85.01	87.5	Alive
	5	Anorexia, general fatigue	8	900	6	51.96	51.96	Alive with lung metastases
	6	Fever > 38℃	7	900	6	86.37	86.37	Alive with regional lymph node metastases
	7	Fever > 38℃	7-9	600-1100	4-8	71.40	71.40	Alive
PEG(+)	1	Fever > 38℃	5	3500	7	74.34	74.37	Alive

RDI and laboratory data

Table 5 presents the detailed data for the calculation of the RDIs of each agent. As expected, the RDIs of the PEG(+) group were significantly higher than those of the PEG(-) group. The P values for docetaxel and cyclophosphamide were 0.0074 and 0.0331, respectively. Thus, the effectiveness of pegfilgrastim in maintaining a high RDI was verified.

**Table 5:** Detailed data for the calculation of dose intensity Both actual and standard doses indicate the total amount of agents administered during the four courses of chemotherapy. The actual and standard times were determined as the interval from day 1 of the 1st course to the same day 1 of the fourth course. The values of the table are expressed by the mean with standard deviation excluding the “standard time.” The significant difference was identified in “relative dose intensity” between the PEG(-) and PEG(+) groups. The P-values of each pair, * and †, in docetaxel and cyclophosphamide are 0.0074 and 0.0331, respectively (Mann-Whitney U test).

-	Docetaxel		Cyclophosphamide	
	PEG(-)	PEG(+)	PEG(-)	PEG(+)
Actual dose (mg)	433.8 ± 58.4	449.1 ± 46.0	3489.7 ± 453.3	3608.0 ± 367.9
Actual time (week)	9.7 ± 1.3	9.2 ± 0.6	9.7 ± 1.3	9.2 ± 0.6
Actual dose intensity (mg/week)	45.2 ± 8.8	48.9 ± 6.8	364.2 ± 69.8	392.9 ± 54.6
Standard dose (mg)	459.9 ± 37.4	460.1 ± 34.3	3679.6 ± 299.7	3681.0 ± 275.1
Standard time (week)	9	9	9	9
Standard dose intensity (mg/week)	51.1 ± 4.1	51.1 ± 3.8	408.8 ± 33.3	409.0 ± 30.5
Relative dose intensity (%)	87.7 ± 14.0*	94.5 ± 8.6^†^	88.2 ± 14.0*	94.8 ± 8.3^†^

**Figure 1:**
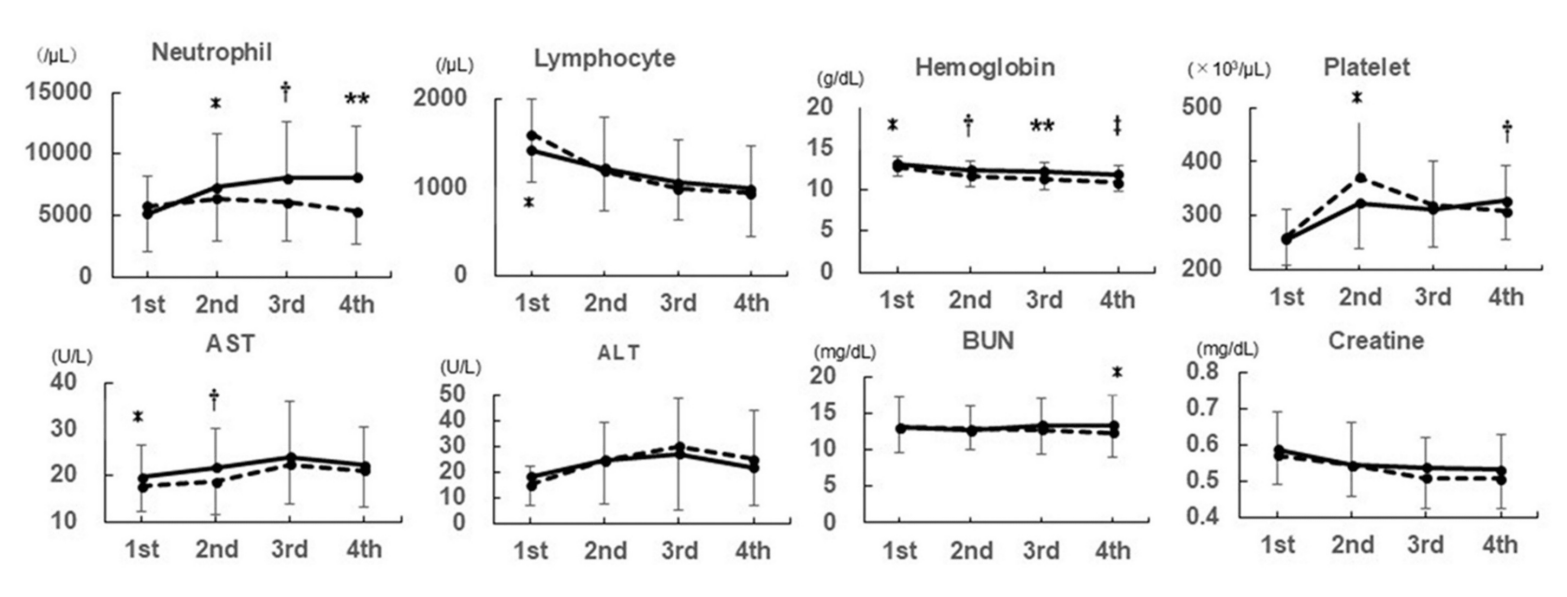
Trends of laboratory tests *,†,**,‡ indicate significant difference (P < 0.005) between the PEG(-) and PEG(+) groups in each course of chemotherapy. Solid and dotted lines indicate PEG(-) and PEG(+), respectively.

The trends in laboratory test results during TC chemotherapy are shown in Figure 1. The evaluated factors included neutrophils, platelets, hemoglobin, aspartate aminotransferase (AST), alanine aminotransferase (ALT), blood urea nitrogen, and creatinine (Figure 1). The neutrophil count in the PEG(-) group was significantly higher than that in the PEG(+) group in each cycle and increased significantly in each cycle. The lymphocyte count decreased gradually in each cycle; however, no significant difference was observed between the two groups, excluding the first cycle. The hemoglobin level during chemotherapy in the PEG(+) group decreased gradually, and the difference between the PEG(-) and PEG(+) groups at the fourth cycle was 1.02 g/dL, indicating a statistically significant difference. The hemoglobin levels in the PEG(-) and PEG(+) groups at the fourth cycle were 11.92 and 10.90 g/dL, respectively.

DFS

**Figure 2:**
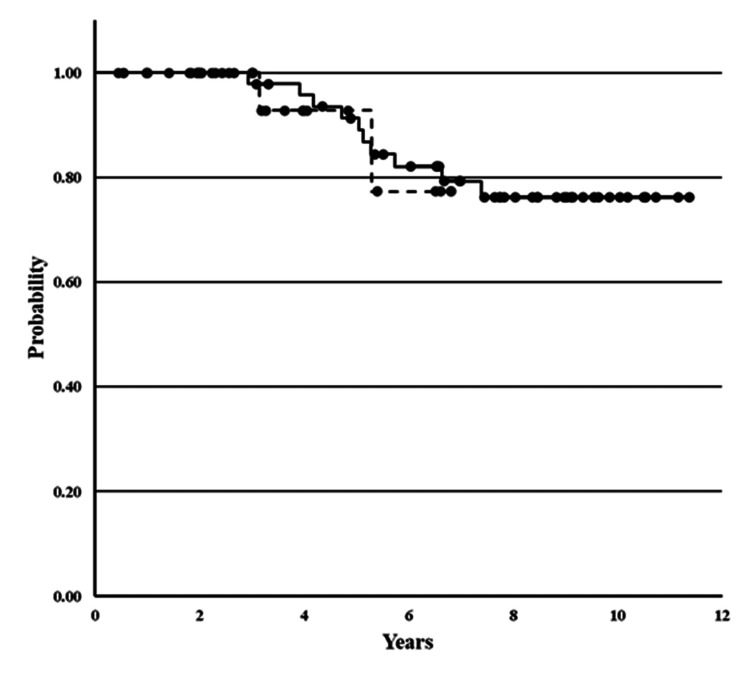
Disease-free survival rate of the PEG(-) and PEG(+) groups Solid and dotted curves indicate PEG(-) and PEG(+), respectively. No significant differences were observed between the two groups using a log-rank test.

Thirteen patients, 10 (20.4%) in the PEG(-) group and three (8.3%) in the PEG(+) group, experienced primary breast cancer recurrence. Two (4.1%) patients of the PEG(-) group died because of recurrence. Analyses of the 13 patients are presented in Table 6. Six (12.2%) and two (4.1%) patients were lost from the follow-up in the PEG(-) and PEG(+) groups, respectively. The mean DFS period of the PEG(-) and PEG(+) groups and the Kaplan-Meier curve are shown in Table 6 and Figure 2, respectively. Because the number of events was small, the difference in DFS rates was not significant.

**Table 6:** Summary of the patients recurred and disease-free survival of all the patients Fisher's exact test was applied for the statistical analyses of recurrence sites.

	PEG(-)	PEG(+)	P-value
No. of patients	10 (20.4%)	3 (7.7%)	-
Recurrence sites			0.422
Local sites, lymph nodes	4	1	
Bone	2	2	
Lung, pleura	3	0	
Liver	2	1	
Ovary	1	0	
RDI (median, range)			
DTX	100.00 (51.96-100)	91.51 (65.13-100)	0.865
CPA	100.00 (51.96-100)	91.30 (67.26-100)	1.000
Disease-free survival period (years, mean±standard error)	9.92 ± 0.40	6.32 ± 0.31	0.7773
95% confidence interval, years	9.12 - 10.72	5.69 - 6.94	

## Discussion

The administration of pegfilgrastim significantly reduced the risk of FN development with acceptable adverse events. The chemotherapy RDI of patients who received prophylactic pegfilgrastim was significantly higher than that of those who were administered conventional filgrastim at the physician’s discretion. The advantages of pegfilgrastim in TC chemotherapy were, therefore, appropriately demonstrated using real-world data from a single institution. 

After confirmation of the superiority in DFS and overall survival (OS) of TC chemotherapy compared to AC chemotherapy [3,4], TC chemotherapy has been introduced to patients with early-stage breast cancer as a postoperative adjuvant chemotherapy. Together with the prevalence of TC chemotherapy, the prophylaxis of FN during this regimen has also been discussed, and G-CSF administration plays an important role in the prevention of FN [10]. In such instances, the prophylactic use of pegfilgrastim produced excellent efficacy in the prevention of FN [11]. Pegfilgrastim is prepared using pegylation technology applied to a recombinant methionyl form of human G-CSF, filgrastim, and by covalently linking a 20-kDa polyethylene glycol molecule to filgrastim. Pegfilgrastim has a longer half-life and lower elimination rate than filgrastim, resulting in an elevated serum concentration over time [12,13].

Maintaining a high RDI during chemotherapy with acceptable adverse events is beneficial for prolonging DFS and OS. Lu Zhang et al. demonstrated that among ER+/PR+, HER2- patients, an RDI of 85% was the cut-off point at which a low RDI was significantly associated with worse OS [14]. In our series, the mean value of RDIs for each agent was higher than 85% (Table 4). This may be beneficial for achieving a better prognosis (Table 5, Figure 2). However, higher RDI may result in higher toxicity rates. No severe adverse events were observed. Tsuboi et al. reported that pegfilgrastim-induced bone pain develops, particularly in younger patients (age < 55 years) [15]. Although we could not identify those symptoms in the clinical setting, the patient diagnosis might be confused with neuralgia, arthralgia, and myalgia, which are also induced by docetaxel administration. The number of patients with Grade 1/2 neuralgia was 19 (38.7%) and 25 (69.4%) in the PEG(-) and PEG(+) groups, respectively (P = 0.008). Grade 2 neuralgia on CTCAE indicates “moderate pain or limiting instrumental activity of daily life (ADL).” Although the level of neuralgic pain ranged between Grade 1 or 2, limitations of ADLs were induced by pegfilgrastim administration. Hoshina et al. reported a case of G-CSF-associated aortitis [16]. The frequency of this disease is reported to be 0.47% in all cases of G-CSF administration. Its etiology is excessive stimulation of inflammatory cytokine production [17]. However, we did not find such cases in our study.

Figure 1 shows the trends in laboratory test results during the four courses of chemotherapy. The neutrophil counts of the PEG(-) group were significantly higher than those of the PEG(+) group. This may have resulted from the elongation of the washout period and dose reduction, as indicated by the decrease in the RDIs in the PEG(-) group. Lymphocyte counts in both groups decreased gradually for every treatment course. Collovà et al. analyzed the lymphocyte trend in patients with breast cancer undergoing dose-dense chemotherapy with pegfilgrastim and reported that B (CD19+, CD20+) and early B lymphocyte subsets (CD20+/CD38+) decreased significantly during chemotherapy, whereas T lymphocyte subsets did not present significant changes, except for a decrease in T helper (CD4+) cells. Immature T lymphocytes (CD4+/CD8+ subset) increased compared to the baseline, and both dendritic cells (CD11c+) and NK cells (CD56+) increased compared to healthy controls [18]. These results indicated that the immune system of patients with breast cancer was maintained during chemotherapy with pegfilgrastim.

Among the other factors evaluated during chemotherapy, the hemoglobin level of the PEG(+) group significantly decreased. The difference between the two groups at the fourth cycle was small, 1.02 g/dL, and the mean PEG(+) level was > 10 g/dL, which was classified as Grade 1 on the CTCAE. This phenomenon may be due to myelosuppression caused by intensive chemotherapy in the PEG(+) group. Changes in other biochemical factors are considered biological variations during chemotherapy.

Medical costs associated with cancer treatment are a factor to be considered. In a randomized placebo-controlled clinical trial comparing the incidence of FN during TC chemotherapy between the pegfilgrastim and placebo groups, pegfilgrastim reduced the costs incurred for both chemotherapeutic agents and hospitalization for adverse events, as well as FN [19].

In clinical settings, clinicians always administer broad-spectrum antibiotic agents when the patient is affected with FN. Exposure to antibiotics during chemotherapy might adversely influence the response to chemotherapy through the modulation of intestinal microbiota. This phenomenon is clarified in the chemotherapy with immune checkpoint inhibitors [20]. Chemotherapy-induced gastrointestinal dysbiosis is responsible for the symptoms such as nausea, vomiting, and diarrhea, which frequently develop during chemotherapy [21]. During chemotherapy, maintaining the intestinal microbiota is considered to be essential for keeping the high response of chemotherapy agents and enhancing the safety of treatment. Avoiding the FN onset by pegfilgrastim shows another clinical usefulness.

A limitation of this study is the retrospective nature of the analysis. The sample size is small because this is a single-institutional study. And the bias for patient selection might be contained. However, the patients enrolled in this study were consecutive cases, and neither the mean age nor the age distribution of the two groups showed statistical differences. We understood the patient performance status in the PEG(-) and PEG(+) groups to be equal. The adverse symptoms, excluding “neutrophil count decrease” in Table 3, were retrospectively collected from the medical records. We cannot ignore that only some of the symptoms were recorded in the medical chart. However, symptoms requiring treatment intervention were noted. These assessments should be performed using a checklist for each medical examination. The study was also limited by the short follow-up period and the small number of patients enrolled. We could also not confirm a difference in OS between the two groups because the number of event cases was small. 

## Conclusions

Among 85 patients with stage I or II breast cancer who received TC chemotherapy, pegfilgrastim administration significantly reduced the risk of FN development, with acceptable adverse events. Moreover, the RDI of patients who received prophylactic pegfilgrastim was significantly higher than that of patients who received conventional filgrastim. We could not find a significant difference in the DFS between the two groups of PEG(-) and PEG(-), although we could verify the clinical efficacy of pegfilgrastim in patients receiving myelosuppressive chemotherapies such as TC chemotherapy. The literature research suggested that administration of antibiotics during chemotherapy has an adverse effect on patients through the disruption of intestinal microbiota. The prevention of FN occurrence by pegfilgrastim results in the reduction of antibiotic administration. This might be beneficial clinically for patients on immunosuppressive status. Further accumulation of cases and long-term follow-up is necessary for the analysis of the OS. Pegfilgrastim use might show a benefit for the improvement of OS. A future prospective trial could offer more controlled conditions and reduce the inherent biases associated with the retrospective data of this study.
